# Supplementary material for: NET Formation in Systemic Lupus Erythematosus: Changes during the COVID-19 Pandemic
Source: Cells. 2022 Aug 23;11(17):2619. doi: 10.3390/cells11172619 (PMC9455008; doi:10.3390/cells11172619)
Supplement: Supplementary file 1 [file cells-11-02619-s001.zip › cells-1871334-supplementary.pdf]

Supplementary Figure S1:

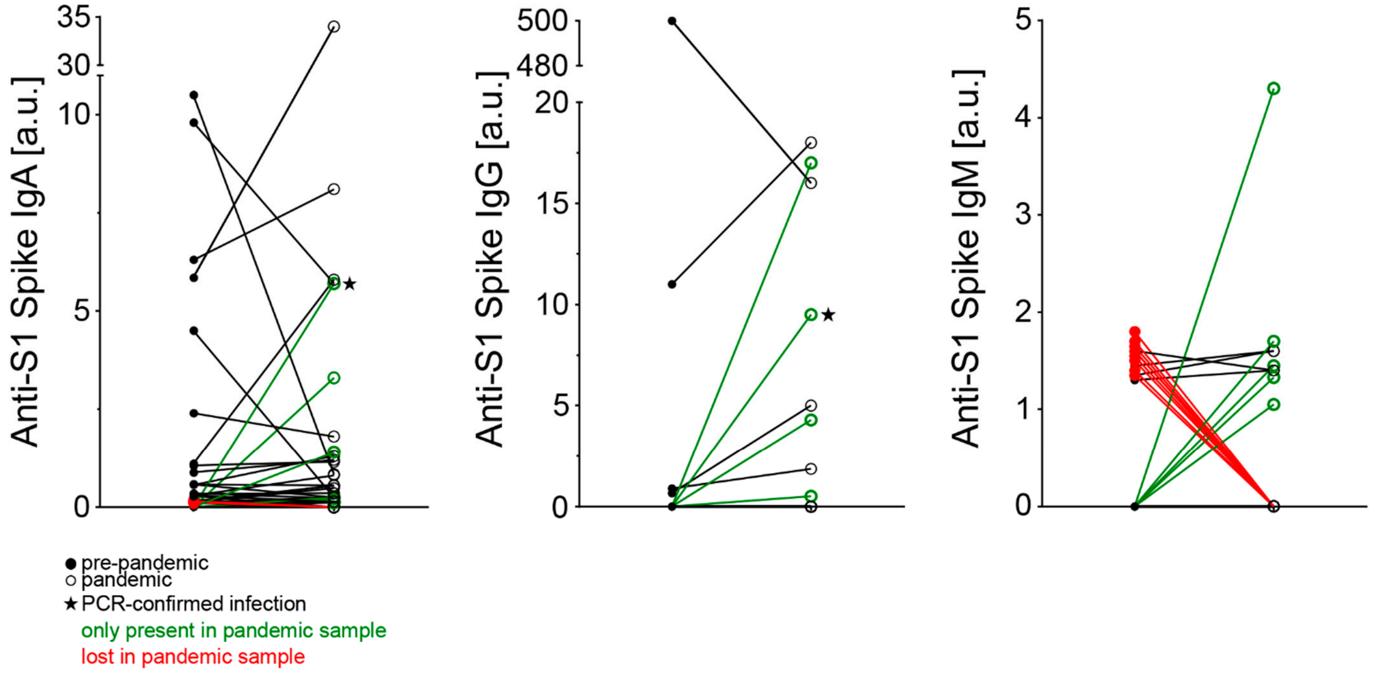

Supplementary Figure S1: Changes in isotypes and levels of COVID antibodies. The presence of antibodies against the S1 subunit of the SARS-CoV-2 spike protein as surrogate marker for infection were assessed by ELISA. Changes of antibody levels among the different isotypes (IgA, IgG, IgM) between the pre-pandemic and pandemic serum samples are depicted. Green lines and empty circles highlight newly emerging antibodies in the pandemic serum samples, whereas red lines and circles show the antibodies that are lost in the pandemic samples. In the samples additionally annotated with a star, the infection with SARS-CoV-2 was additionally confirmed by PCR.

Supplementary Table S1: Changes in antibody levels and isotypes within the same individual inbetween the two cohorts. Antibody levels and isotypes were assessed by ELISA.

Subject	Anti-S1 IgA	Anti-S1 IgG	Anti-S1 IgM
1	+	n.d.	n.d.
2	n.d.	+	n.d.
3	new	n.d.	+
4 x	lost	n.d.	n.d.
5	-	n.d.	lost
6	n.d.	+	n.d.
7	+	new	n.d.
8	-	n.d.	n.d.
9	+	n.d.	n.d.
10	-	n.d.	new

11	new	+	n.d.
12	+	n.d.	n.d.
13	-	n.d.	+
14	-	n.d.	n.d.
15	-	n.d.	n.d.
16	-	n.d.	n.d.
17	n.d.	-	new
18	n.d.	n.d.	+
19	+	n.d.	lost
20 x	similar	n.d.	n.d.
21	+	n.d.	n.d.
22	+	+	n.d.
23	+	n.d.	n.d.
24	+	n.d.	-
25	+	n.d.	lost
26	-	n.d.	new
27	+	n.d.	lost
28	+	n.d.	n.d.

x SARS-CoV-2 infection confirmed by PCR
+ increase in antibody levels
- decrease in antibody levels
n.d. not detected
new antibody isotype only detected in pandemic sample
lost antibody isotype only detected in pre-pandemic sample
Similar similar levels of antibody isotype in both samples

Supplementary Figure S2:

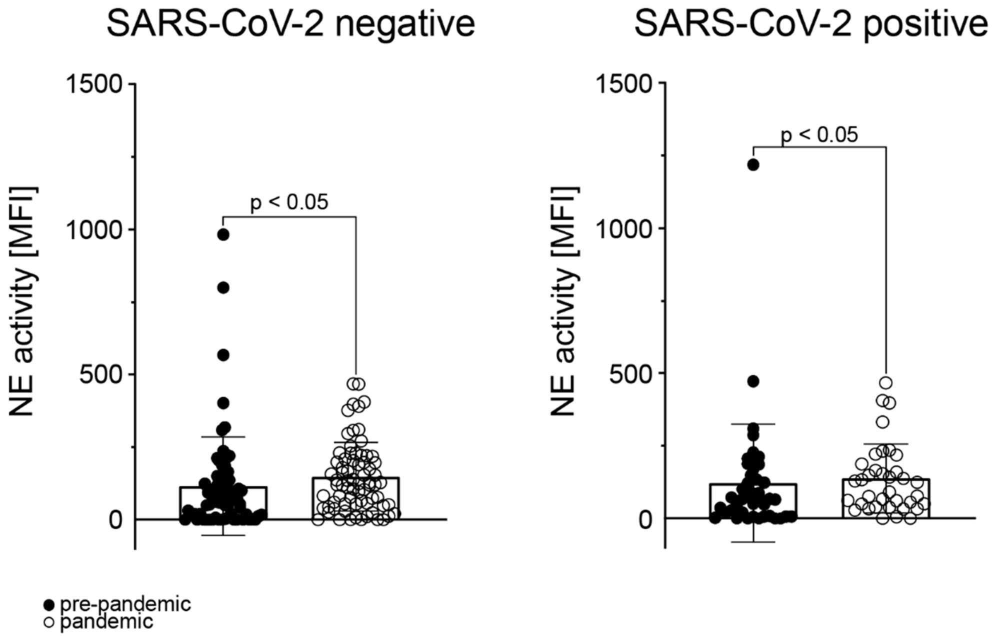

Supplementary Figure S2: Comparison of NE activity in SARS-CoV-2 negative and positive samples. The activity of NE was assessed by fluorescent substrate conversion (mean fluorescence intensity (MFI)) in samples from both cohorts showing either no signs of serological exposure to SARS-CoV-2 (SARS-CoV-2 negative) or had a PCR confirmed infection/were positive for antibodies (IgA, IgM and/or IgG) against SARS-CoV-2 (SARS-CoV-2) as indicated in Table 2. Statistical significance was calculated using Wilcoxon matched-pairs signed rank test.
